# Speed Breeding of Soybean by Using 22 h Photoperiod Increases Photochemical Efficiency of Pods and Produces Six Generations Per Year

**DOI:** 10.1111/ppl.70511

**Published:** 2025-09-12

**Authors:** Seher Bahar Aciksoz, Shellie Wall, Stuart James Lucas, Mustafa Atilla Yazıcı, Tracy Lawson

**Affiliations:** ^1^ Sabanci University Nanotechnology Research and Application Center (SUNUM) Turkey; ^2^ School of Life Sciences University of Essex Colchester UK; ^3^ School of Animal Rural and Environmental Sciences Nottingham Trent University Nottingham UK; ^4^ Department of Plant Biology Institute for Genomic Biology Urbana IL US; ^5^ Faculty of Engineering and Natural Sciences Sabanci University Istanbul Turkey

**Keywords:** long‐day photoperiod, nonfoliar photosynthesis, soybean, speed breeding, stress tolerance

## Abstract

Fast generation cycling of plants has the potential to overcome the bottleneck of traditional breeding programmes, which often require several years to achieve the desired outcomes. Recent speed breeding methodologies have reduced generation times in both short‐ and long‐day species by optimizing environmental conditions. However, protocols for short‐day plants impose a constant short‐day photoperiod throughout the entire life cycle, even though plants could benefit from extended light exposure. Here, we report a speed breeding scheme for soybean ( *Glycine max* ) based on a long‐day photoperiod of 22 h (LD‐22 h) applied upon flowering initiation (stage R1) using light‐emitting diodes (LEDs) with a cool white (6000 K) and red light (660 nm) spectrum at 550 μmol/(m^2^s) photosynthetic photon flux at canopy level. We also outline an immature seed germination technique for early harvested green seeds collected from speed‐breeding plants that markedly increased the germination rate. Combining these methods allowed our soybean speed breeding system to acquire a 92% germination rate from 58‐day‐old seeds, enabling six generations y^−1^ compared to typically only 1–3 using standard approaches. The impact of long photoperiods on soybean leaf and pod photochemical efficiency was examined. Although photosynthetic capacity (*Vc*_max_, *J*_max_, and *A*_max_) was significantly lower in leaves grown under LD‐22 h photoperiod, seed production was unaffected, while PSII operating efficiency (*F*_*q*_ *′/F*_*m*_ *′)* in pods was markedly higher under LD‐22 h compared to the SD‐10 h photoperiod. Implementing our post‐flowering long photoperiod conditions followed by an enhanced germination technique could facilitate rapid breeding for soybeans and be adapted for use with other photoperiod‐sensitive short‐day crops.

## 1 Introduction

Plant breeding is one of the key elements for developing disease resistant, climate resilient, and nutritious crops with higher yields. Breeding of cultivar lines typically takes 4–6 generations of growth to test multiple traits of interest and develop genetically stable homozygous lines and a minimum of 8–10 years before the release of a new improved cultivar (Alahmad et al. 2018). The time taken for seed generation presents a significant bottleneck for plant breeders aiming at enhancing crop genetics. Hence, any method facilitating additional generation cycles would result in a cumulative increase in genetic progress. Speed breeding was first introduced for long‐day crops such as spring bread wheat, durum wheat, barley (Zheng et al. 2013), pea, canola, and chickpea by using a 22 h prolonged photoperiod during the entire plant growth (Watson et al. 2018; Ghosh et al. 2018). However, this concept cannot be applied to short‐day photosensitive crops that require a longer than critical night length for initiation of flowering, which varies considerably among species (Jackson 2009).

Soybean ( *Glycine max* ), a facultative short‐day plant, is the most cultivated crop among legumes and the fourth crop in the world after wheat, rice, and maize (FAOSTAT database) and therefore one of the most important dicot crops worldwide. It is increasingly cultivated for its protein and oil content for human consumption and as a primary protein source in animal feed (Dong et al. 2014; Pratap et al. 2012), as well as being used for industrial and pharmaceutical materials (Yamada et al. 2012). Therefore, enhancing soybean production is crucial to meet the increasing demand for the food and materials produced from this key crop. Several speed breeding approaches have been reported for soybean, including the use of plant regulators, such as cytokinin and cold stress (Mobini et al. 2020; Gallino et al. 2022), far‐red wavelengths (> 700 nm) early flower induction (Childs et al. 1997; Craig and Runkle 2013), red and blue growth light spectra rather than the full white spectrum (Harrison et al. 2021), CO_2_ supplementation (Nagatoshi and Fujita 2019), early harvesting of immature seeds (Fang et al. 2021), far‐red deprived, blue‐light enriched spectrum (Jähne et al. 2020) and LED systems instead of sodium lamps (Lee et al. 2023). All these techniques have reduced regeneration time and produced four to five generations of soybean plants annually. However, using these methods also has several disadvantages, including the relatively high cost of spectrum adjustable LED lamps (making them unsuitable for plant research facilities with limited budgets or large‐scale production), as well as the need for infrastructure to allow CO_2_ supplementation, along with the increasing costs of CO_2_ gas. Concurrently adding plant growth regulators to the growth medium is not only costly but also laborious. Finding speed breeding solutions that overcome some of these cost implications, without negatively impacting yield, would therefore greatly benefit the plant breeding community.

Photosynthetic capacity is one of the most important parameters that determines yield potential and the basis of crop productivity. Chlorophyll fluorescence measurements not only provide a rapid and non‐destructive tool for determining photosynthetic efficiency, but these measurements can also be used as an indicator of overall plant health (Baker 2008). Most studies to date focus on measuring photosynthesis in leaves (Bhatta et al. 2021; Hussain et al. 2019); however, increasing attention has also been paid to nonfoliar material such as ears, pods, and fruits (Simkin et al. 2020; Herritt et al. 2020; Lawson and Milliken 2023). Nonfoliar photosynthesis, either as net photosynthesis or via refixing internally respired carbon (internal CO_2_ recycling), can contribute significantly to the overall carbon gain of plants, e.g., accounting for up to 60% of the total carbon requirement of reproductive organs (Aschan and Pfanz 2003). Cho et al. (2023) showed that soybean seed and pod photosynthesis provide up to 9% of the canopy daily carbon gain and account for up to 14% of the mature seed weight. In legumes, green photosynthetic pod tissues cover the seeds, and electron transport rates in these tissues have been shown to vary depending on plant species and canopy architecture, which ultimately determines the amount of light received (Allen et al. 2009; Tschiersch et al. 2011). The individual contributions of green nonfoliar pods and leaves to photosynthetic processes in soybean canopies grown under 22 h daylength, as in our speed breeding system, had not been determined prior to this work.

Here, we present a 22 h photoperiod‐based speed breeding system for soybean that produces six consecutive generations within a year. This new method requires a photoperiod adjustment to 22 h once soybeans flower (R1), in conjunction with an integration of a green seed dormancy breaking technique to generate plants from immature harvested seeds, that dramatically shortens the seed‐to‐seed life cycle of soybeans. We further aimed to explore the effect of an extended photoperiod on photochemical efficiency in soybean pods by comparison with leaves, and we gained insights into the importance of nonfoliar‐pod photosynthesis under long‐day speed breeding systems. High‐throughput chlorophyll fluorescence analysis showed that *F*_*q*_′/*F*_*m*_′ of soybean pods receiving 22 h light (exposed upon flowering) was significantly higher than pods from the 10 h light conditions.

## 2 Materials and Methods

### 2.1 Plant Growth Conditions

Seeds of soybean plants ( *Glycine max* (L.) Merr.) were transplanted into 7 × 7 × 8 cm pots, fitting 30 pots in one tray. A commercial potting soil (BioBizz light mixture) was used as the growth medium. All experiments were completely randomized on each tray, using a full factorial design. One soybean plant was grown per pot. For germination rate and soluble carbohydrate experiments, soybean plants were initially established under a short‐day 12‐h photoperiod and upon anthesis (R1 stage ~24 DAS), the photoperiod was adjusted into three groups: long‐day 22 h light (LD‐22 h), long‐day 16 h light (LD‐16 h), and short‐day 12 h light (SD‐12 h). SD‐12 h daylength was used as the control photoperiod. Cool white (6000 K) and red light (660 nm) light‐emitting diodes (LEDs) with spectral power distribution of 6:1, respectively, were used for the experiments at a total photosynthetic photon flux of 550 μmol m^−2^ s^−1^ at the canopy level (Figure S1a). Temperature was set to 24°C day/night; relative humidity varied from 80% to almost 90%. The experiments were conducted in a plant growth cabinet under controlled climatic conditions. We recorded the flowering time when the first blossom emerged. Green seeds were harvested at three time points: 58 days after sowing (DAS), 65 DAS, and 77 DAS to test the germination rate (Figure S1b). Germination rate was calculated as the germinated seed number divided by the total number of seeds per plant.

Photosynthetic rate and measurements of chlorophyll fluorescence were performed on soybean grown under a short‐day 10‐h photoperiod, and once R1 flowering stage was reached, the photoperiod was adjusted to either long‐day 22 h (LD‐22 h) or short‐day 10 h (SD‐10 h). Plants were grown in the same conditions as described above, except that humidity was maintained around 60%. All the plants were watered daily from below.

### 2.2 Green Seed Dormancy Breaking for Early‐Harvested Seeds

**FIGURE 1:**
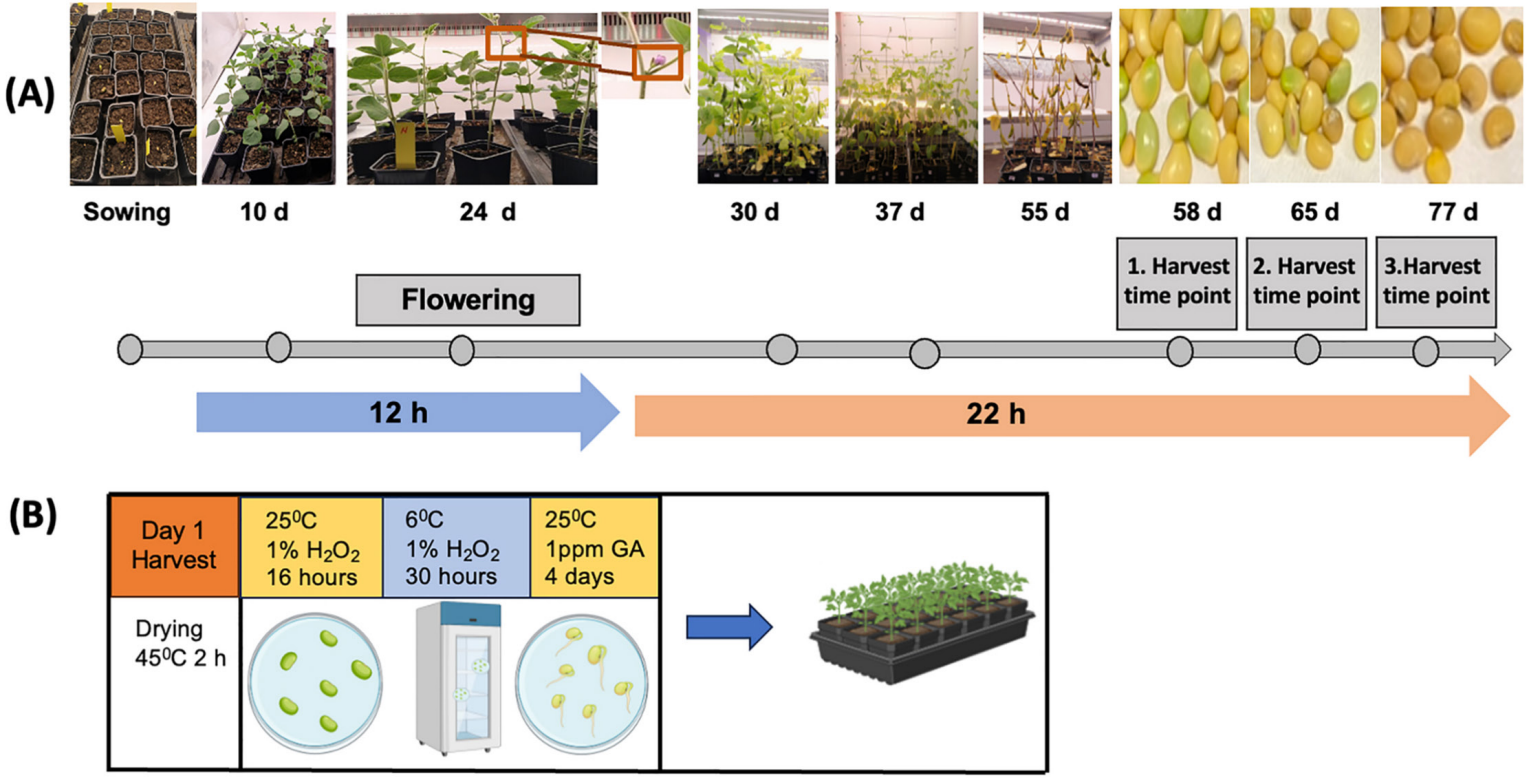
Soybean speed breeding scheme from sowing to harvest and subsequent germination method. (A) 12 h daylength was used till anthesis (R1 stage‐around 24 DAS), afterwards the photoperiod was extended to 22 h until harvest. Seeds were collected from plants at harvest time point of days 58, 65, and 77. (B) Harvested seeds were subjected to the green seed dormancy breaking method.

Watering was interrupted 7 days before harvest to accelerate the ripening process. The harvested pods were kept in paper bags and dried at 45°C for 2 h to reduce the moisture content. Upon threshing, green seeds were treated with 1% hydrogen peroxide (H_2_O_2_) solution at 25°C room temperature for 16 to 17 h in petri dishes, then cold‐treated in the same solution at 6°C for 30 h in a dark cold room. The seeds were then kept under dark conditions at 25°C and treated with 1 ppm gibberellic acid (GA3; Sigma, cat. no. G7645). The germination took place on filter paper under dark conditions at room temperature (25°C). Seeds were observed for germination daily, and the radicle count was conducted on day 7. Radicle protrusion was counted as physiological germination, and the missing emerged radicle was considered as not germinated. This new germination technique is summarized in Table 1 and illustrated in Figure 1B. After germination, the sprouting seeds were sown in pots, and shoot lengths were observed when the seedlings were 15 days old.

**TABLE 1:** Speed seed dormancy breaking and cold treatment applied to immature soybean seeds.

Day 1	Harvest the pods
	Drying at 45°C for 2 h and hand threshing
	Treatment with H_2_O_2_ (1%) at 25°C for 16–17 h
Day 2	Treatment with H_2_O_2_ (1%) at 6°C for 30 h
Day 3	Germination with gibberellic acid (1 ppm) at 25°C
Day 7	Green seeds are germinating, count the germination rate

### 2.3 Chlorophyll Fluorescence Imaging of Plants

Chlorophyll fluorescence imaging was used to determine the dark adapted maximum quantum yield of PSII photochemistry (*F*_*v*_ */F*_*m*_) and the light operating efficiency of PSII photochemistry (*F*_*q*_ *′/F*_*m*_ *′*) utilizing a Fluorimager chlorophyll fluorescence imaging system (Technologica Ltd). The measurements were taken at room temperature (22°C ± 2°C) between 9 a.m. and 3 p.m. Intact plants were analyzed with attached pods and leaves from SD‐10 h and LD‐22 h photoperiods (22 h photoperiod started on 30 DAS‐upon anthesis) at 48, 49, and 50 DAS. All plants were dark adapted for 1 h before being placed in the imager. Minimal fluorescence (*F*_*o*_) was determined using less than 1 μmol m^−2^ s^−1^ PPFD before maximum fluorescence (*F*_*m*_) was determined following a saturating pulse (6354 μmol m^−2^ s^−1^, for 800 ms). These measurements were used to determine images of *F*_*v*_ */F*_*m*_ applying the following equation:

$$F_{v}/F_{m}=\left(F_{m}-F_{o}\right)/F_{m}$$

After that, actinic light was turned on at 800 μmol m^−2^ s^−1^ and the induction of photosynthetic efficiency (*F*_*q*_ *′/F*_*m*_ *′)* captured with saturating pulses taken every 3 min for 60 min from the light and dark adapted measurements maximum operating efficiency in the light (*F*_*v*_ *′/F*_*m*_ *′*) was determined using the following equation:

$$\left({F_{m}}^{\prime }-{F_{o}}^{\prime }\right)/{F_{m}}^{\prime }$$

### 2.4 Gas Exchange Measurements

The response of *A* to changes in intercellular CO_2_ concentrations (C*i*) was determined on the youngest 4th trifoliate at V4 stage on 38 DAS plants using a LICOR (Li‐6800; LI‐COR) with a 6 cm^2^ cuvette. Plants were grown under a SD‐10 photoperiod initially, and once anthesis occurred on 30 DAS, the photoperiod was altered to either LD‐22 or SD‐10 h photoperiod until measurements at 38 DAS. All measurements were performed at an air temperature of 26°C, relative humidity of 65%, leaf VPD of 1.1 ± 0.1 kPa; the leaf cuvette CO_2_ concentration was kept at 400 μmol mol^−1^ and a flow rate of air through the system of 500 μmols^−1^. Once photosynthesis was stabilized at 400 μmol mol^−1^ CO_2_, the value decreased to 300, 200, 150, 75, 50, and next increased to 500, 700, 850, 1000, 1200, 1400, 1600, 1800, then returned to an initial value of 400 μmol mol^−1^. Photosynthesis was measured at each CO_2_ level after 3 min. All measurements were conducted between 9 a.m. and 3 p.m. to minimize the effect of any diurnal or circadian rhythms. The maximum saturated CO_2_ assimilation rate (*A*_*max*_), the maximum velocity of Rubisco carboxylation (*Vc*_*max*_) and the maximum rate of electron transport demand for RuBP regeneration (*J*_*max*_) were calculated from the *A*/*C*_*i*_ data using equations from Caemmer and Farquhar et al. (1980) as demonstrated by (Sharkey et al. 2007), and fitted using the fitaci function in the R package *plantecophys* (Duursma 2015).

### 2.5 Soluble Carbohydrate Determination

Soluble carbohydrate analysis was performed on leaves harvested at 58 DAS by using the anthrone method (Yemm and Wills 1954). Samples were dried in an oven at 45°C and analyzed individually. Dried and milled plant samples were extracted with 80% ethanol (1:100 w:v), the suspensions were centrifuged at 15,000 *g* for 20 min, and the supernatants were collected. 4 mL of anthrone reagent with sulfuric acid was added to 250 μL of the supernatant. The mixture was incubated in a water bath at 95°C for 11 min. When the samples cooled down, the absorbance was read at 620 nm. D‐glucose was used for the calibration of the spectrophotometer. The soluble carbohydrate concentration was calculated from the specific weights used for analysis.

### 2.6 Statistical Analysis

One‐way analysis of variance (ANOVA) and comparisons of means using Tukey's honestly significant difference test at *p* < 0.05 level were performed. The responses of dry weight, total soluble carbohydrate, and seed germination rates were analyzed by principal component analysis in R Studio version 2023.09.1 + 494.

## 3 Results

### 3.1 Acceleration of Generation Advancement in Soybean Plants

**FIGURE 2:**
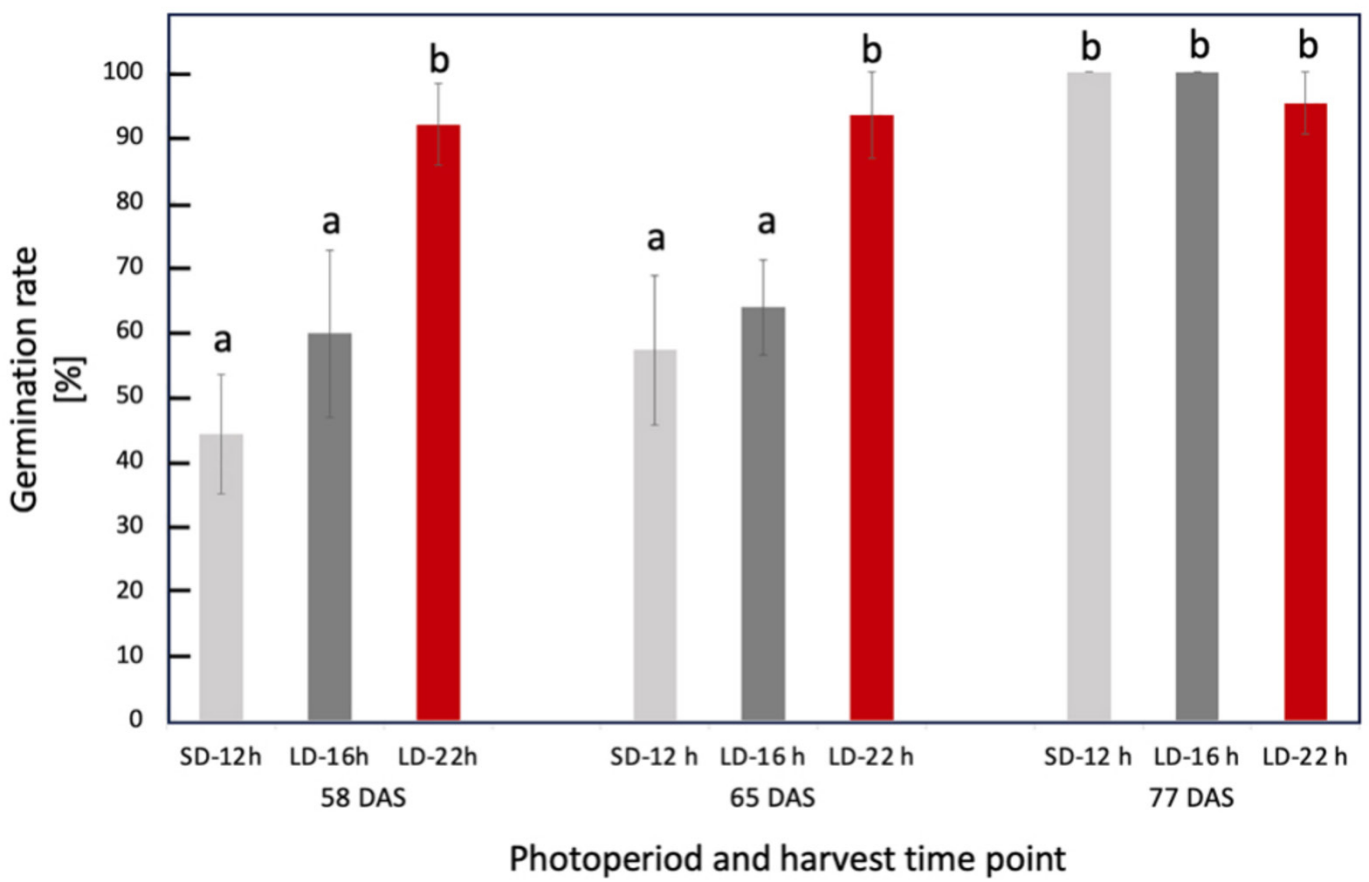
Seed germination rates of individual plants grown under 12 h daylength during entire growth period (SD‐12 h) and extended to 16 h daylength (LD‐16 h) and 22 h (LD‐22 h) daylength after anthesis and harvested at time points of day 58, 65, and 77. Values are means of 8 to 10 independent replicates. Different letters indicate significant differences according to one‐way ANOVA and Tukey post hoc test (*p <* 0.05).

Soybean seeds were sown under cool white LED light (6000 K) supplemented with red LEDs (660 nm) at a white: red ratio of 6:1, and a total light intensity of 550 μmol m^−2^ s^−1^ at canopy level. When these light conditions were applied with a 12 h photoperiod, flowering occurred at 24 days after sowing (DAS), significantly accelerating seed setting (Figures S1c, S2), which averages 56 DAS in the field (Naeve 2011). We optimized the photoperiod condition upon onset of flowering to a long‐day daylength of 22 h, which accelerated seed maturity, enabling early harvest on day 58. By collecting green seeds from speed‐grown plants and applying our germination technique to these premature seeds, a germination rate of more than 90% on the 7th day of germination treatment was achieved (Figure 2). An overview of the photoperiod optimization for soybean speed breeding and the subsequent post‐harvest green seed germination enhancement approach is provided in Figure 1.

We examined the critical harvest time point to achieve the highest germination rate of soybean seeds from 58, 65, and 77 day old plants grown under light exposures of SD‐12 h, LD‐16 h, and LD‐22 h. When seeds were harvested from 58‐day‐old plants, the germination rate (92% ± 6.25%) was significantly higher in the plants grown under the longest photoperiod (LD‐22 h), while lower germination rates were observed for the LD‐16 h (57% ± 12.8%) and SD‐12 (44% ± 9.21%) photoperiods Figure 2. A similar trend was observed for seeds harvested from 65‐day‐old plants, in which the LD‐22 h germination rate was significantly higher at 93% ± 6.66%, compared to the LD‐16 h and SD‐12 photoperiods which had lower values of 63% ± 7.29% and 57% ± 11.55%, respectively Figure 2. When seeds were harvested from 77‐day‐old plants, the photoperiod did not affect seed germination, with all three photoperiods having germination rates close to 100% (Figure 2). In order to test for seedling establishment, germinated seeds were transferred to seed trays and cultivated in the growth chamber using the pre‐anthesis light conditions. By 15 days of transfer, all seedlings had reached heights of 20–25 cm with no differences observed between photoperiod groups, at which point measurements were terminated.

**FIGURE 3:**
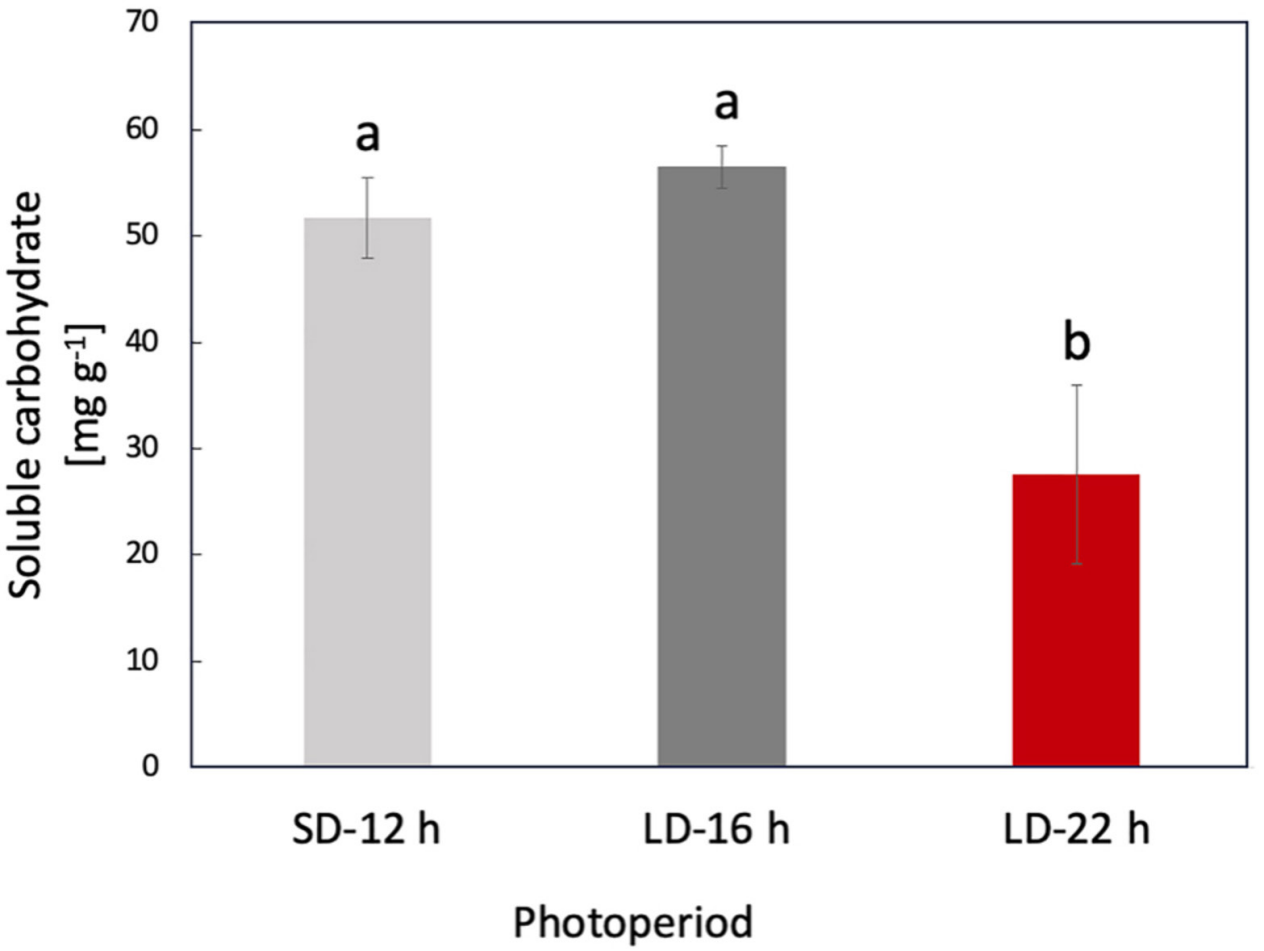
Effect of photoperiod on soluble carbohydrate concentration in leaves (mg g^−1^) at 58 DAS. All plants were grown in a SD‐12 h (light gray) photoperiod until flowering, after which treatment groups were subjected to long day photoperiods of LD‐16 h (dark gray) or LD‐22 h (red), while control group was kept in short day conditions (SD‐12 h). Values are three to four independent replicates. Different letters indicate significant differences between means according to one‐way ANOVA and Tukey post hoc test (*p* < 0.05).

We evaluated the effect of SD‐12 h, LD‐16 h, and LD‐22 h photoperiods on soluble carbohydrate accumulation in leaves harvested at day 58. Increasing the photoperiod from SD‐12 to LD‐16 did not significantly affect soluble carbohydrate levels, whereas increasing the photoperiod further from LD‐16 to LD‐22 h significantly decreased a level of soluble carbohydrate levels to less than half of that accumulated in 58‐day‐old plants grown under SD‐12 h or LD‐16 h (Figure 3).

### 3.2 Photosynthetic Capacity Measured as Photosynthesis as a Function of Intracellular CO_2_ Response Curves (*A*/*C*_*i*_)

**FIGURE 4:**
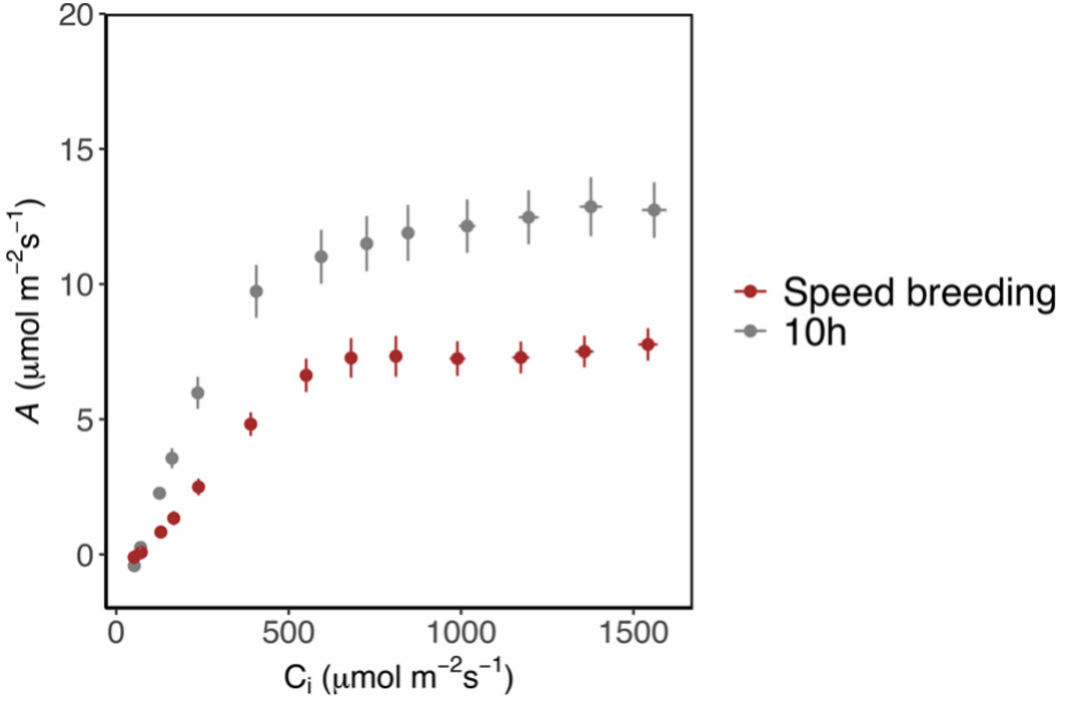
Net CO_2_ assimilation response (*A*) of grown soybeans to increasing intercellular CO_2_ results (*Ci*) from 150 to 1800 ppm for the speed breeding LD‐22 h (22 h light period applied after flowering) and SD‐10 h photoperiod (10 h light period during entire growth period). Light intensity was 1600 μmol m^−2^ s^−1^ photosynthetically active photon flux density (PPFD). Error bars represent mean SE ± (*n* = 5–6). Treatments: SD‐10 h and LD‐22 h.

To determine the impact of photoperiod conditions on photosynthesis capacity in leaves, *A*/*C*_*i*_ curves (assimilation rate (*A*) measured as a function of internal CO_2_ concentration) were generated from soybeans grown under a SD‐10 h and LD‐22 h photoperiod. As expected, *A* showed a typical hyperbolic response with increasing *C*_*i*_, with *A* initially increasing linearly until photosynthesis was saturated and steady state was reached (*A*_max_; Figure 4).

**FIGURE 5:**
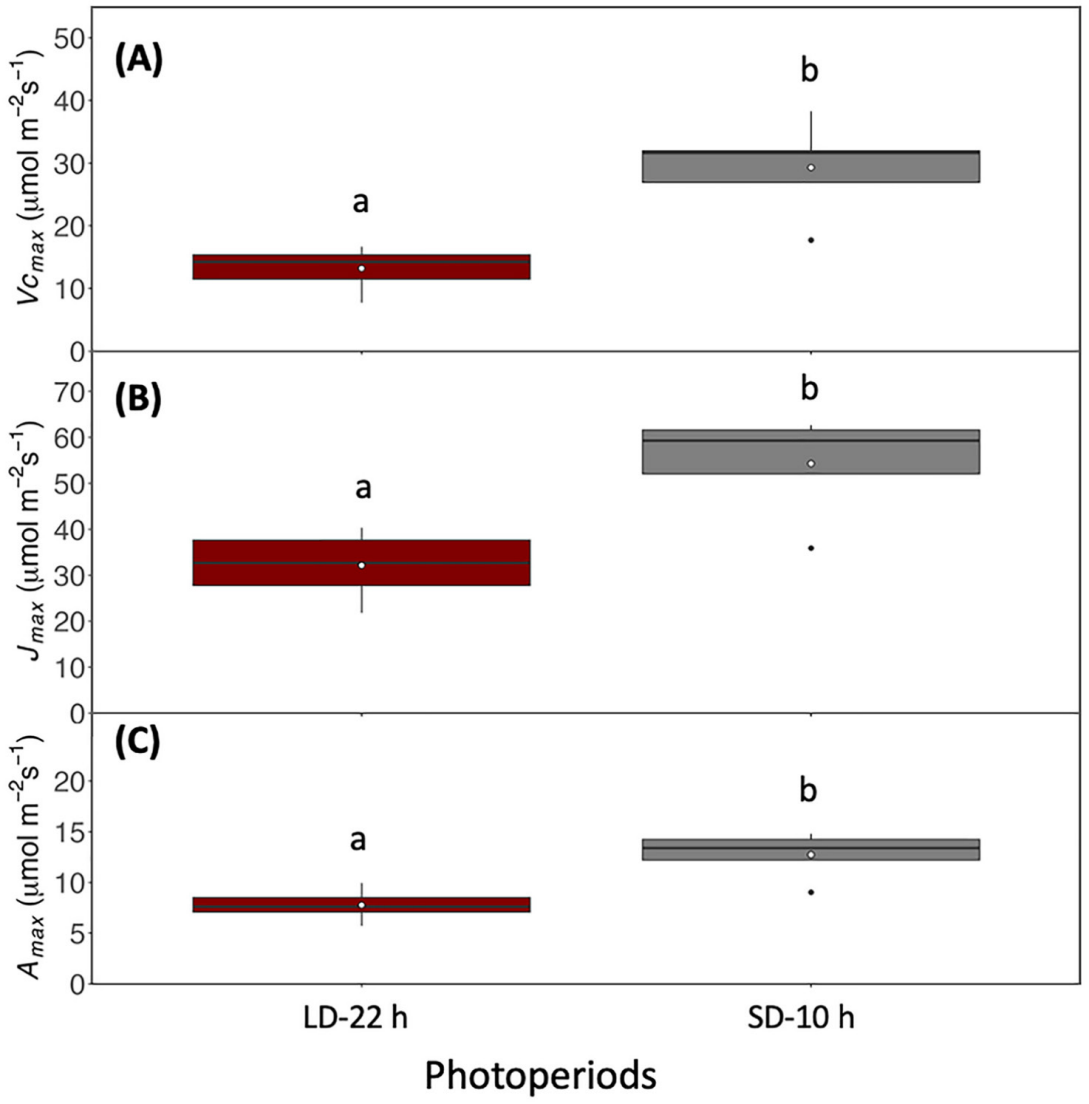
Boxplots showing variation and means (white dots) of maximum rate of carboxylation (*Vc*_max_). (A) maximum rate of electron transport (*J*_*max*_). (B) and CO_2_ saturated rate of photosynthesis (*A*_*max*_). (C) of soybean under SD‐10 and LD‐22 h growth conditions. Initial 10 h photoperiod was switched to 22 h photoperiod after flowering in LD‐22 h treatment and SD‐10 h plants were grown under a consistent 10 h photoperiod. Different lower case letters represent statistically significant differences (*p* < 0.05) between means of each cultivar using the results of a Tukey post hoc test following a two‐way analysis of variance (ANOVA; *n* = 5–6).

Comparing the *A*/*C*_*i*_ curves, plants that were maintained at SD‐10 h reached higher *A* than those transferred to LD‐22 h after flowering; this difference was statistically significant at *C*_*i*_ values above c.150 μmol mol^−1^ (*p* < 0.05, Figure 4). This suggests that the long photoperiod reduced the photosynthetic capacity of leaves; however, this did not cause a reduction in seed dry biomass (Figure S3). *A*_max_ in leaves at the SD‐10 h photoperiod was significantly higher than in leaves at the LD‐22 h photoperiod, suggesting a higher photosynthetic capacity (Figure 5C). In vivo estimates of maximum velocity of Rubisco carboxylation (*Vc*_max_) and the maximum rate of electron transport demand for RuBP regeneration (*J*_*max*_) calculated from *A*/*C*_*i*_ curves were both significantly higher in leaves from SD‐10 h compared to LD‐22 h photoperiod (Figure 5A,B). Leaves of LD‐22 h showed significant reductions of 37%, 55%, and 40%, respectively, in *A*_*max*_, *Vc*_max_, and *J*_*max*_ compared to SD‐10 h (*p* < 0.05; Figure 5).

### 3.3 Variation in the Response of PSII in Leaves and Pods of Soybean Plants

In order to evaluate the effect of daylength on photosynthetic efficiency of leaves and pods photosynthesis under SD‐10 h and LD‐22 h photoperiods, we used chlorophyll fluorescence imaging. The maximum PSII efficiency (*F*_*v*_ */F*_*m*_) of the leaves from the SD‐10 h photoperiod was 0.704, which was significantly lower at 0.4 in the LD‐22 h leaves, whilst the *F*_*v*_ */F*_*m*_ of the pods was not affected by the photoperiods (*p* < 0.05; Table 2).

**TABLE 2:** Effects of short day 10 h photoperiod during entire growth period (SD‐10 h) and 22 h photoperiod after anthesis (LD‐22 h) on the maximum PSII efficiency (*F*_v_/*F*_m_) of soybean leaves and pods measured at 48, 49, and 50 DAS.

Treatment	Tissue	*F*_*v*_ */F*_*m*_	
SD‐10 h	Leaf	0.704 ± 0.011	b
LD‐22 h		0.400 ± 0.013	c
SD‐10 h	Pod	0.781 ± 0.007	a
LD‐22 h		0.780 ± 0.004	a

*Note:* Different letters indicate significant differences between means according to one‐way ANOVA and Tukey post hoc test (*p* < 0.05) (*n* = 3 to 6).

**FIGURE 6:**
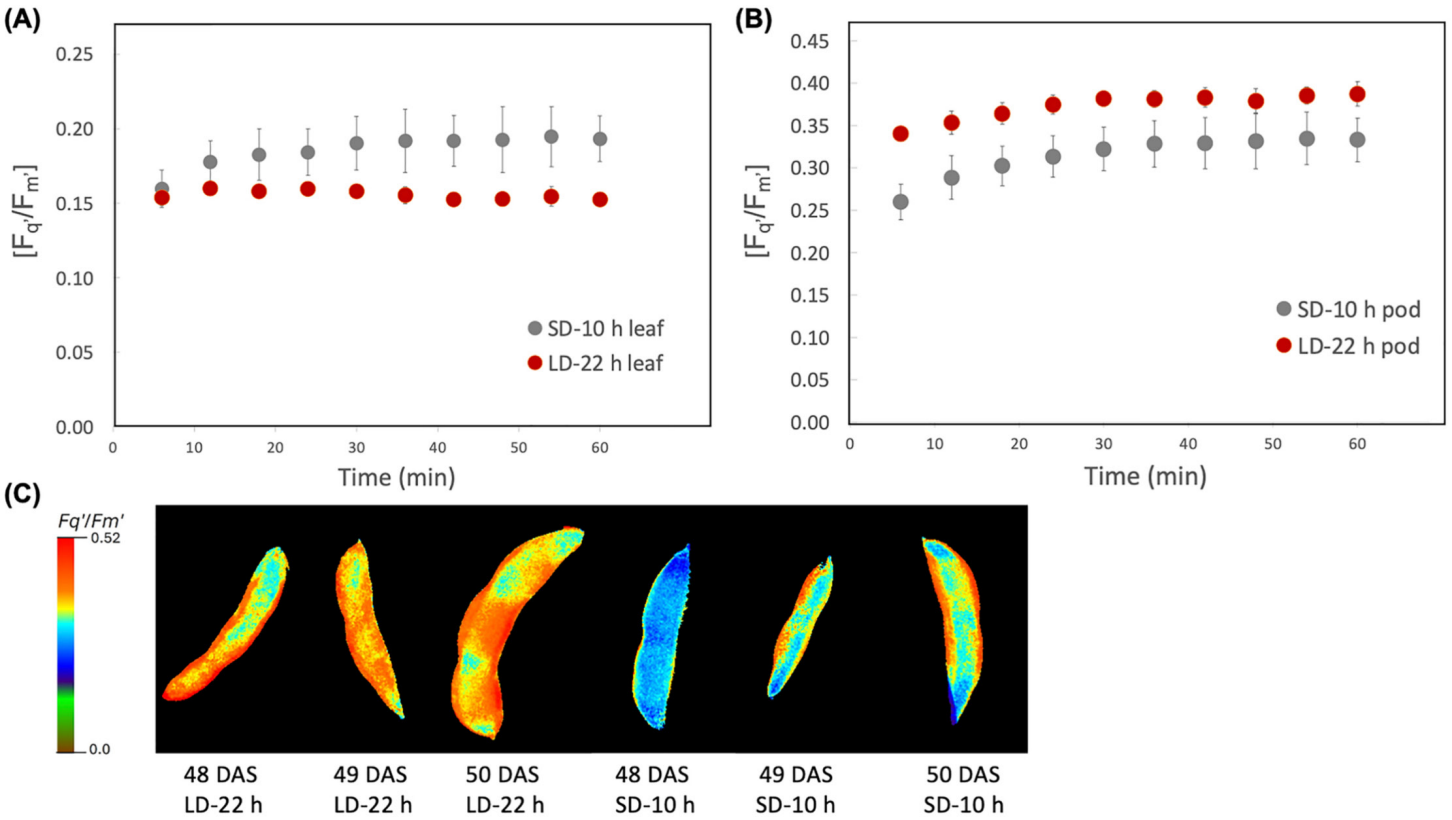
Determination of photosynthetic capacity in soybean leaves and pods using fluorescence imaging. PSII operating efficiency (*F*_*q*_ *′/F*_*m*_ *′*) of soybean leaves grown under SD‐10 h (blue circle) and LD‐22 h (orange circle) photoperiods (A) soybean pods grown under SD‐10 h and LD‐22 h photoperiods. (B) After a dark adaption period of 1 h, the plant material was subjected to an actinic light intensity of 800 μmol m^−2^ s^−1^ at time zero and measurements of the operating efficiency of PSII photochemistry (*F*_*q*_ *′/F*_*m*_ *′*) were taken every 3 min for 60 min. (C) Chlorophyll fluorescence (CF) image of PSII operating efficiency (*F*_*q*_ *′/F*_*m*_ *′*) of pods. Plants were grown under 10 h photoperiod during entire growth period in SD‐10 h light treatment and daylength was extended to 22 h after anthesis in LD‐22 h light treatment. Data are reported as means ± SE of measurements at 48, 49, and 50 DAS (*n* = 3–6).

We compared the operating efficiency of PSII in the light (*F*_*q*_ *′/F*_*m*_ *′*) between the leaves and pods from the photoperiod treatments. Leaves of the SD‐10 h photoperiod had significantly higher operating efficiency of PSII (*F*_*q*_ *′/F*_*m*_ *′*) compared to plant leaves from LD‐22 h (Figure 6A). On the contrary, plant pods exposed to the LD‐22 h photoperiod exhibited significantly higher *F*_*q*_ *′/F*_*m*_ *′* compared to those under the SD‐10 h photoperiod (*p* < 0.05; Figure 6B).

## 4 Discussion

### 4.1 Speed Breeding of Soybean Plants

**FIGURE 7:**
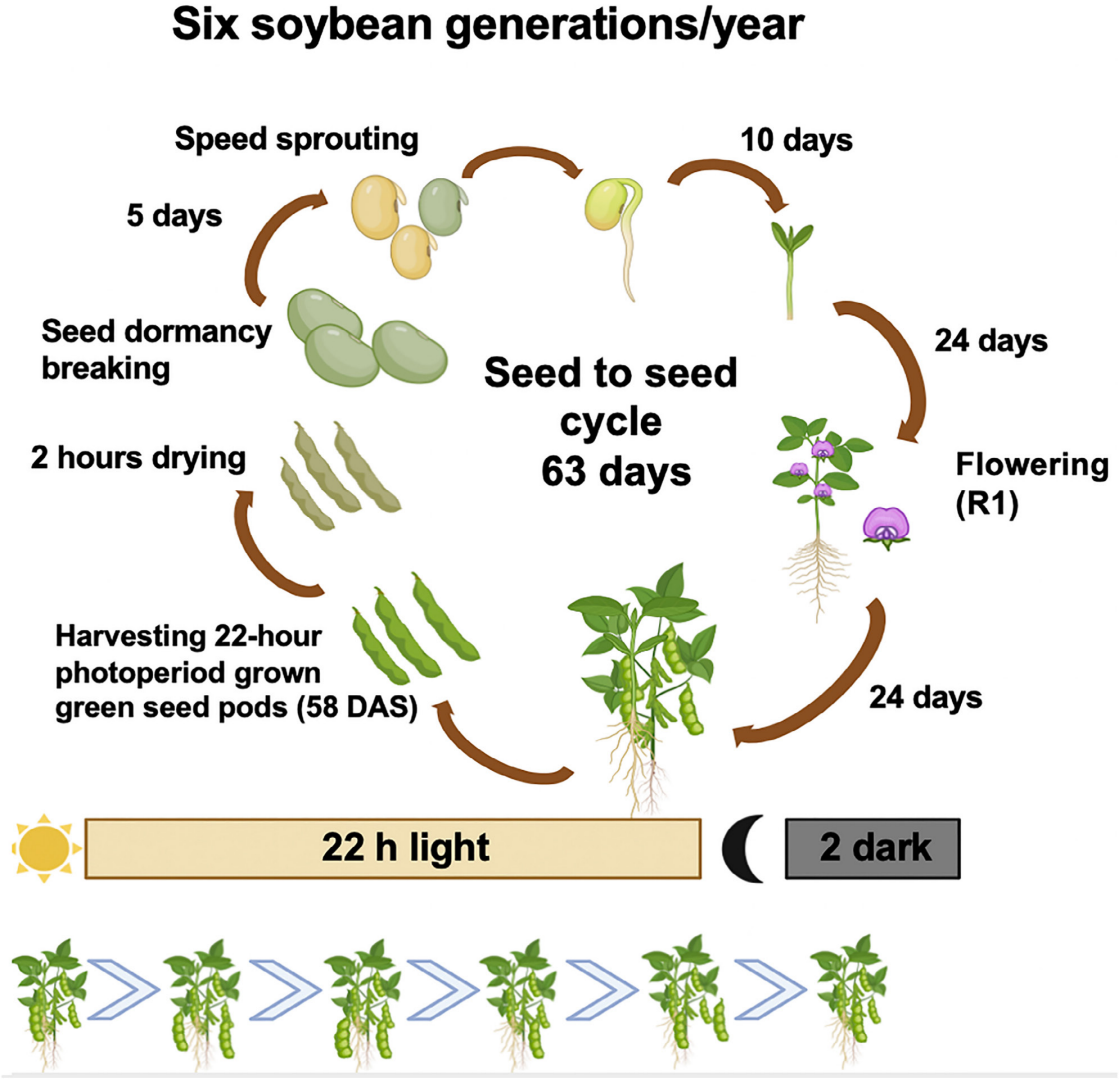
Illustration of the seed‐to‐seed soybean speed breeding cycle presented in this study. Our method includes a 22 h light/2 h dark cycle that is applied post‐anthesis, harvesting of premature seeds at 58 DAS and dormancy‐breaking cold treatment. This reduces the seed‐to‐seed generation time from ~120 days in field conditions to 63 days, allowing almost six soybean generations per annum. This figure was created in BioRender.com.

Here, we present a soybean speed breeding method that reduces the generation time of soybean to 63 days after sowing and can produce up to six consecutive generations in a year compared with 2–3 generations in a standard greenhouse or growth chamber regime (Figure 7). The protocol used initially employs a 12 h photoperiod until the flowering stage (R1), and upon flowering, the photoperiod is extended to 22 h until harvest. In addition, after harvest, a dormancy‐breaking cold treatment is applied to the green seeds, increasing the germination rate (Figure 1). Previous soybean speed breeding studies suggested that long‐day photoperiods of speed breeding methods cannot be applied to short‐day plants due to inhibition of flowering (Ghosh et al. 2018). Thus, this is the first study to highlight that post‐flowering long‐day photoperiod (22 h) could be effective for short‐day crops to speed up seed maturity. Jähne et al. (2020) showed that increasing light intensity above 1000 μmol m^−2^ s^−1^ led to 2 days of early flowering in soybeans; however, soybean leaf photosynthesis rates have been reported to plateau at 500 μmol m^−2^ s^−1^ (Long et al. 2006). Thus, excess absorbed light energy was wasted as heat dissipation, consequently decreasing light use efficiency (Niyogi and Truong 2013; Zhu et al. 2008). Using CO_2_ supplementation and high temperatures of 30°C to reduce the soybean generation time, as used by Nagatoshi and Fujita (2019) were not considered as part of our method because these additions come with significantly higher costs, such as special equipment and greater energy use. The presented protocol recommends using a light intensity of ~500 μmol m^−2^ s^−1^ (without exceeding soybean photosynthetic capacity of CO_2_ reduction) and a temperature of 24°C, which is sufficient to shorten the cycle duration.

Seed production, stem and leaf dry weights (g per plant) of soybean plants were not significantly affected by photoperiods of SD‐12 h, LD‐16 h, and LD‐22 h, highlighting an opportunity to use extended long‐day photoperiods on soybeans without reducing seed and plant material production. The parameters presented in our speed breeding approach can be optimized for a variety of other agronomically significant photoperiod‐sensitive short‐day cultivars, accessions, and species and can be integrated into rapid generation systems in breeding programs. Wheat grown in a growth cabinet compared to glasshouse conditions showed a higher and more stable photosynthesis rate over a 10 h period (Bhatta et al. 2021), demonstrating supplemental artificial lighting systems offer advantages for sustained photosynthesis by a consistent delivery of illuminance amounts. Our method of applying the 22‐h light period from flowering onwards additionally may be expanded to indoor farming systems for photoperiod‐sensitive plant production purposes, low budget breeding facilities, and plant research in private companies, as generic LED lamps were used as supplemental lighting, without a requirement for additional equipment such as CO_2_ supplementation or cultivar‐specific tailored light spectra for plant growth. However, mildly stressful growth conditions often result in limited seed yield and tillering; therefore, biomass thresholds for each crop variety should be considered. In particular, the F1 generation should be grown at a lower planting density to obtain a sufficient amount of seeds for the F2 generation.

### 4.2 22 h Photoperiod Decreases Leaf Photosynthetic Capacity

We further investigated the interaction between photoperiod, leaf photosynthesis, and leaf soluble carbohydrate accumulation to explain whether the accelerated plant growth cycle is due to enhanced photosynthetic activity. When keeping light intensity constant, extending the photoperiod is expected to lead to enhanced photosynthesis as the plants receive an increased daily light integral. However, our photosynthetic measurements revealed that extending photoperiod from SD‐10 h to LD‐22 h led to significant decreases in *A*_*max*_, *J*_*max*_, and *Vc*_max_ and soluble carbohydrate concentration, which indicates reduced photosynthetic capacity under the long‐day photoperiod (Figure 5). Sawada et al. (1989) showed that soybean leaves under 24 h daylength had significantly lower photosynthetic rates, lower activation ratio of Rubisco, and lower sucrose and starch content compared to plants grown in a 10 h photoperiod. In tomato, extending the photoperiod over 12 h decreased the net photosynthetic rate (Dorais et al. 1996) and the level of soluble carbohydrates was halved when the photoperiod was extended from 16 h to 23.5 h (Jensen and Veierskov 1998). In potato, whenever the photoperiod was increased from 12 h up to 24 h, *A*_*net*_ was greatly decreased, also showing the inability of plants to maintain high photosynthesis levels under long photoperiods due to tissue damage (Stutte et al. 1996). In the present study, although application of the post‐flowering LD‐22 h photoperiod decreased the soluble carbohydrate levels in the leaf, LD‐22 h seeds had the highest germination rates compared to those of SD‐12 h and LD‐16 h seeds at day 58 (Figure 2). Sun et al. (2024) showed that genes related to starch, sucrose, carbohydrate, and energy metabolism were repressed under long‐day conditions in soybeans. The effect of post‐flowering photoperiod extension on soybean leaf and pod photosynthetic traits and leaf soluble carbohydrate metabolism has not been reported previously. Despite reductions in leaf photosynthesis, extended light did not provoke decreases in leaves and seed dry biomass of LD‐22 h plants in our study. This is thought to be due to the use of a moderate light intensity to limit tissue damage. The lack of relationship between leaf photosynthesis and biomass accumulation could be due to the involvement of other processes such as respiration (Lambers et al. 1983) and soybean pod and seed photosynthesis (Burgess and Degen 2023), which also determine yield. Furthermore, measurements of photosynthetic capacity are rarely achieved during the growing period and therefore do not always provide a representation of the realized rates of photosynthetic carbon assimilation over the growing period (Lawson et al. 2012). Additionally, measurements were made on individual leaves and therefore do not represent the entire plant canopy (Zelitch 1982; Campbell et al. 1986).

### 4.3 Increased Quantum Yield of PSII in 22 h Pods

Chlorophyll fluorescence is a non‐intrusive method that reflects the light energy absorption and utilization by photosystem II (PSII; Schreiber et al. 1995). *F*_*v*_ */F*_*m*_ is an important indicator of the maximum efficiency of the primary light energy transfer within the PSII antenna (Maxwell and Johnson 2000; Murchie and Lawson 2013), and can be used as a monitor of “stress” with values of *F*_*v*_ */F*_*m*_ lower than 0.832 (Björkman and Demmig 1987; Murchie and Lawson 2013) indicating a degree of stress or photoinhibition (Baker and Rosenqvist 2004). In this study, *F*_*v*_ */F*_*m*_ values of LD‐22 h leaves were significantly lower than SD‐10 h leaves, suggesting that these leaves suffer from some photoinhibition and/or severe light‐induced inhibition. This reduction was also observed in the operating efficiency of PSII photochemistry (*F*_*q*_ *′/F*_*m*_ *′)* in LD‐22 h leaves compared to SD‐10 h leaves. These observations indicate LD‐22 h causes a reduction in the photosynthetic capacity reflected by the reduced maximum quantum yield of PSII (*F*_*v*_ */F*_*m*_). Similarly, photoinhibition was observed in cucumbers (Shibaeva and Markovskaya 2013) and coriander (Wang et al. 2024) grown in long days (> 20 h photoperiod), whilst 24 h light treatment increased photoinhibition in both cucumber and tomato (Shibaeva et al. 2022).

However, there were no significant differences in *F*_*v*_ */F*_*m*_ values of pods between LD‐22 h and SD‐10 h, showing that pods had a better photosynthetic performance than leaves under long day photoperiod stress. Although most studies focus on photosynthesis and carbon assimilation capacity in foliar tissue, recent studies have demonstrated that photoassimilates provided by nonfoliar tissues contribute significantly to whole‐plant carbon assimilation (Lawson and Milliken 2023; Cho et al. 2023; Sanchez‐Bragado et al. 2016). It has been shown that when leaves were detached in wheat, photosynthesis in nonfoliar ear tissues represents a major source of carbon fixation and can contribute to grain filling by up to 40% (Maydup et al. 2010). Under drought stress, ear photosynthesis becomes a more important source than leaf photosynthesis in grain filling of durum wheat (Tambussi et al. 2007) and barley (Bort et al. 1994). Such compensations in photosynthetic activity in the remaining organs also occurred when foliage was shaded (Chanishvili et al. 2005). It has been reported that in some plants nonfoliar tissues, photosynthetic carbon gain is supported by high CO_2_ released from mitochondrial respiration that is then re‐fixed by Rubisco (Aschan and Pfanz 2003; Millar et al. 2011). Soybean pods have green tissue that covers the seeds, in which Rubisco has been shown to be active and contributes to photosynthesis (Allen et al. 2009). Electron transport rate (ETR) in photosystem II has also been demonstrated in green soybean seeds (Borisjuk et al. 2005). Our results suggest that the increase in PSII operating efficiency (*F*_*q*_ *′/F*_*m*_ *′)* in pods can compensate for the reduction in photosynthetic activity in leaves under the LD‐22 h photoperiod treatment. We propose that increased pod photosynthetic activity may also contribute to the higher germination rates of seeds from these pods (Figure 2). Seed germination is a highly energy‐intensive process, and photosynthetic light absorption in green seeds generates ATP and reduced NADPH that could help to meet the energy demands (Celdran et al. 2015; Shackira et al. 2022). We suggest that further investigations and a greater understanding of the physiological mechanisms of seed or pod photosynthesis are essential. Knowledge of diurnal adaptations and biosynthetic activity of green seed germination will help to develop novel strategies for crop improvement. The protocol we developed here identifies the effectiveness of long‐day photoperiod application in soybean plants, which can be further optimized to account for genotypic variations and be extended to other short‐day crops, offering a universal framework for reducing generation times and accelerating breeding outcomes of short‐day crops.

## Author Contributions

S.B.A. conceived the study and performed the experiments. S.B.A., S.W., T.L. designed the research. S.B.A. and S.W. analyzed the data. S.B.A. and T.L. wrote the paper with inputs from all co‐authors. All authors have read and agreed to the published version of the manuscript.

## Conflicts of Interest

The authors declare no conflicts of interest.

## Supporting information


**Figure S1:** Effects of the speed breeding protocol on the development of plants and seeds. (a) Emission spectrum of LED light modules; the two channels were combined at 100% intensity. (b) Effect of photoperiods on seeds from harvest time points (day 58, 65, 77) (c) Impact of photoperiods on soybean morphology at 30 DAS. Scale bar = 20 cm.
**Figure S2:** Extended photoperiods result in senescence even in early growth stages. Images show the development of 30‐day‐old soybean grown under SD‐12 h (A), LD‐16 h (B), and LD‐22 h (C) photoperiod in growth chambers. Scale bar = 10 cm.
**Figure S3:** Seed, stem, and pod dry weights of plants grown under SD‐12 h, LD‐16 h, LD‐22 h photoperiods and harvested at time points of day 58, 65, and 77. Values are means of eight to 10 independent replicates. Different letters indicate significant differences according to one‐way ANOVA and Tukey post hoc test (*p <* 0.05).

## Data Availability

The data that support the findings of this study are available from the corresponding author upon a reasonable request.
