# Supplementary material for: Speed Breeding of Soybean by Using 22 h Photoperiod Increases Photochemical Efficiency of Pods and Produces Six Generations Per Year
Source: Physiol Plant. 2025 Sep 12;177(5):e70511. doi: 10.1111/ppl.70511 (PMC12426813; doi:10.1111/ppl.70511)
Supplement: Supplementary file 1 — Figure S1: Effects of the speed breeding protocol on the development of plants and seeds. (a) Emission spectrum of LED light modules; the two channels were combined at 100% intensity. (b) Effect of photoperiods on seeds from harvest time points (day 58, 65, 77) (c) Impact of photoperiods on soybean morphology at 30 DAS. Scale bar = 20 cm. Figure S2: Extended photoperiods result in senescence even in early growth stages. Images show the development of 30‐day‐old soybean grown under SD‐12 h (A), LD‐16 h (B), and LD‐22 h (C) photoperiod in growth chambers. Scale bar = 10 cm. Figure S3: Seed, stem, and pod dry weights of plants grown under SD‐12 h, LD‐16 h, LD‐22 h photoperiods and harvested at time points of day 58, 65, and 77. Values are means of eight to 10 independent replicates. Different letters indicate significant differences according to one‐way ANOVA and Tukey post hoc test (p < 0.05). [file PPL-177-e70511-s001.docx]

**Supporting information for “Speed breeding of soybean by using 22 h photoperiod increases photochemical efficiency of pods and generates six cycles per year**


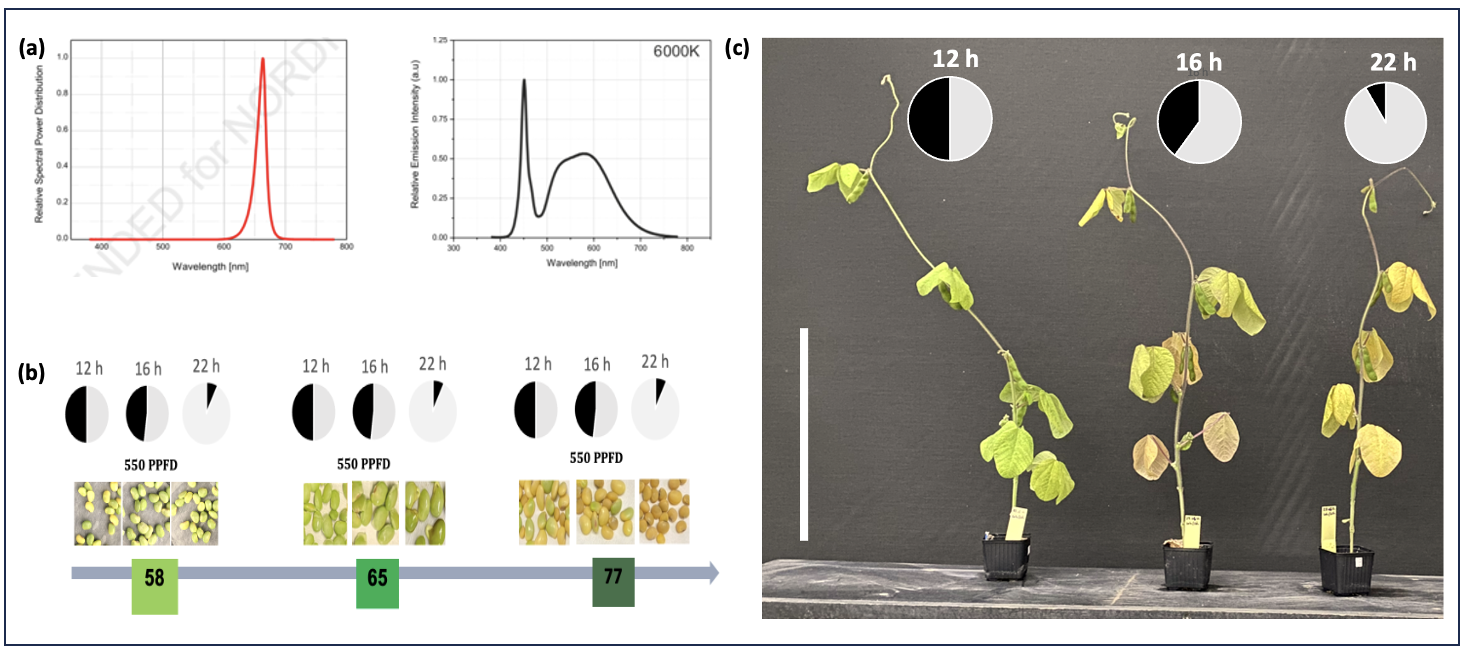


**Figure S1.** Effects of the speed breeding protocol on the development of plants and seeds. (a) Emission spectrum of LED light modules, the two channels were combined at 100% intensity. (b) Effect of photoperiods on seeds from harvest time points (day 58, 65, 77) (c) Impact of photoperiods on soybean morphology at 30 DAS. Scale bar = 20 cm.

**Figure S2.** Extended photoperiods result a senescence even in early growth stages. Images show development of 30-day-old soybean grown under SD-12 h (A), LD-16 h (B) and LD-22 h (C) photoperiod in growth chambers. Scale bar = 10 cm.


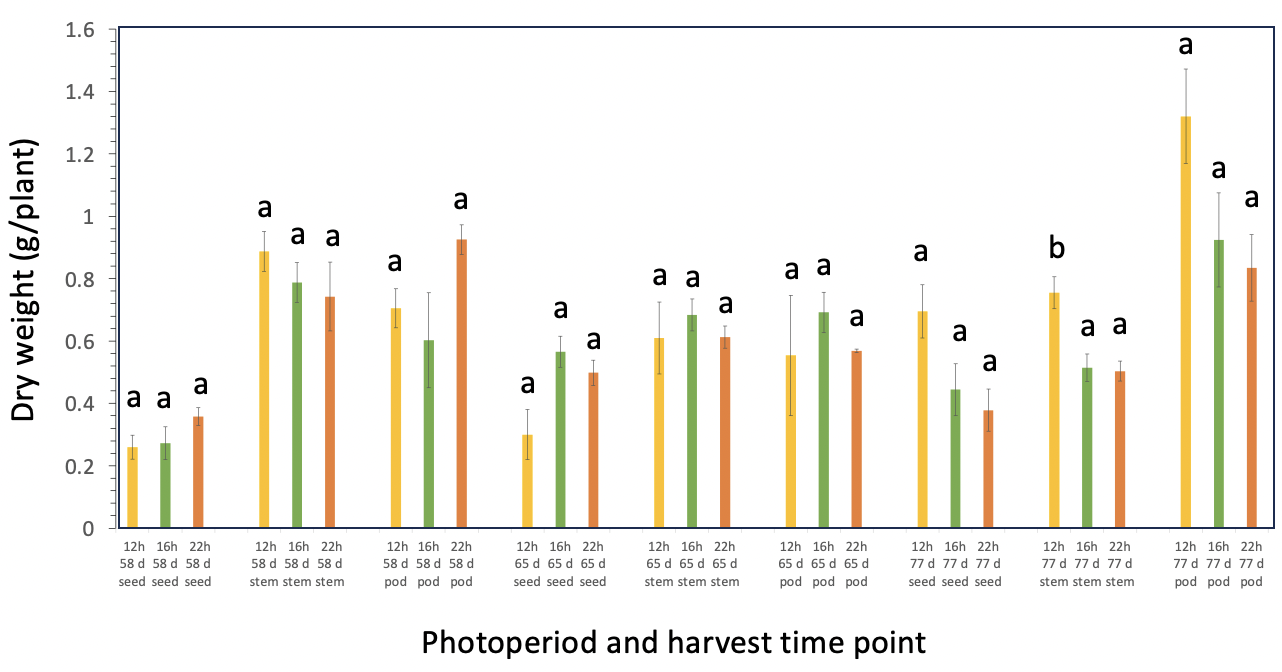
 **Figure S3.** Seed, stem and pod dry weights of plants grown under SD-12 h, LD-16 h, LD-22 h photoperiods and harvested at time points of day 58, 65 and 77. Values are means of eight to ten independent replicates. Different letters indicate significant differences according to one-way ANOVA and Tukey post-hoc test. (P*<*0.05).
